# A triangle study of human, instrument and bioelectronic nose for non-destructive sensing of seafood freshness

**DOI:** 10.1038/s41598-017-19033-y

**Published:** 2018-01-11

**Authors:** Kyung Mi Lee, Manki Son, Ju Hee Kang, Daesan Kim, Seunghun Hong, Tai Hyun Park, Hyang Sook Chun, Shin Sik Choi

**Affiliations:** 1 Department of Food and Nutrition, Myongji University, Yongin, Gyeonggi 449-728 Republic of Korea; 2 Department of Energy Science and Technology, Myongji University, Myongji, Gyeonggi 449-728 Republic of Korea; 3 Interdisciplinary Program for Bioengineering, Seoul National University, Seoul, 151-742 Republic of Korea; 4 Department of Food Science and Technology, Chung-Ang University, Ansung, Kyonggi 456-756 Republic of Korea; 5 Department of Biophysics and Chemical Biology, Seoul National University, Seoul, 151-747 Republic of Korea; 6 Department of Physics and Astronomy and Institute of Applied Physics, Seoul National University, Seoul, 151-742 Republic of Korea; 7 School of Chemical and Biological Engineering, Seoul National University, Seoul, Republic of Korea; 8 Advanced Institutes of Convergence Technology, Suwon, 433-270 Republic of Korea

## Abstract

Because the freshness of seafood determines its consumer preference and food safety, the rapid monitoring of seafood deterioration is considered essential. However, the conventional analysis of seafood deterioration using chromatography instruments and bacterial colony counting depends on time-consuming and food-destructive treatments. In this study, we demonstrate a non-destructive and rapid food freshness monitoring system by a triangular study of sensory evaluation, gas chromatography-mass spectroscopy (GC-MS), and a bioelectronic nose. The sensory evaluation indicated that the acceptability and flavor deteriorated gradually during post-harvest storage (4 °C) for 6 days. The GC-MS analysis recognized the reduction of freshness by detecting a generation of dimethyl sulfide (DMS) from the headspace of oyster in a refrigerator (4 °C) at 4 days post-harvest. However, the bioelectronic nose incorporating human olfactory receptor peptides with the carbon nanotube field-effect transistor sensed trimethylamine (TMA) from the oyster at 2 days post-harvest with suggesting early recognition of oysters’ quality and freshness deterioration. Given that the bacterial species producing DMS or TMA along with toxins were found in the oyster, the bacterial contamination-driven food deterioration is rapidly monitored using the bioelectronic nose with a targeted non-destructive freshness marker.

## Introduction

The assessment of food poison depends on classical instrumental analysis methods including GC (gas chromatography) and HPLC (high performance liquid chromatography) after destructive extraction and preparation of analytes, target toxic compounds by physical and chemical pretreatments of food samples. Specific compounds indicating the progress of food poison are separated and identified through the pretreatment and chromatographic analysis. Biological contaminations of foods have been determined by molecular and biological methods such as PCR (polymerase chain reaction), counting of microorganism colonies, and metagenomic analysis^1^. Foods are composed of wide ranges of ingredients and matrices, and thus these instrumental and biological assays require professional skills and extensive time for sample preparation. The above mentioned analytical methods are ineffective for raw seafood, because the food might be already digested before the safety results are given to consumers.

An oyster (*Crassostrea gigas*) is a popular raw seafood that has long been consumed in many countries. Because the freshness of oyster is critical for safety, as well as sensory qualities including flavor and taste, a precise and rapid monitoring to assess oyster freshness is necessary in food service chains. Conventional methods to determine oyster deterioration are dependent on classical chromatography (GC and HPLC)^2,3^ after time-consuming sample preparation using a solid phase microextraction (SPME)^4,5^ or sensory evaluation conducted by a group of trained panelists. However, these methods are not adequate to real-time and on-site food freshness monitoring that food service chains require for the safety and quality of fresh seafoods.

According to FAOSTAT Database in 2011, 32.80 kg/capita/yr of seafoods were consumed in China, and 21.70 in USA, and 22.90 in the European Union. Japan and Korea showed high consumption levels of 53.70 and 58.10 kg/capita/yr, respectively. In a study by Huss, Reilly, and Ben Embarek (2000), molluscs and fish belong to the highest risk category that can be served as a raw food. Korea’s Consumer Injury Surveillance System (CISS, 2013) reported that seafoods constitute more than 30% of cases in total food poisoning outbreaks. Among the various factors that induce food poison in seafoods^6–10^, the biological contaminations by bacteria, viruses, and parasites, which cause illnesses from mild gastroenteritis to life-threatening diseases such as septicemia^8^. Seafood deterioration leads to not only degradation of seafood muscles but also changes in profile of volatile chemical compounds^11,12^, that are mainly triggered by enzymatic reactions or lipid oxidation processes^13,14^.

Gaseous volatile compounds vaporized from seafoods contribute to the odorant molecules for the indicators of their quality. Trimethylamine (TMA) with a fishy and ammonia-like odor has been used as an index of seafood quality or freshness^15,16^. Indole has also been used as a freshness indicator for shrimp and other crustaceans^17^. However, volatile compounds associated with spoiled seafood have so far rarely been identified. Moreover, volatile compound extraction methods such as steam distillation, purge and trap, simultaneous distillation extraction, dynamic headspace, and vacuum distillation^18^ are time-consuming and labor-intensive procedures because of their small concentrations in foods. Using these extraction techniques, indole, cedrene, TMA, 1,3-diethenyl benzene, 2,5-octadiene, (E,Z)-3,6-nonadien-1-ol, and 1-ethenyl-4-ethyl-benzene were detected and identified from various seafood including oysters, clams, crabs, and prawns^12,19^.

The bioelectronic nose sensors equipped with olfactory receptor protein have been majorly focused on sensing small concentration of odorant molecules rather than detecting volatile organic compounds^20^. In the previous study, the bioelectronic nose that uses human olfactory receptor-derived peptides as primary recognition elements and carbon nanotubes as secondary transducers, has been used for the assessment of seafood freshness^21^. The bioelectronic noses detected a small concentration of TMA that has been known as an odorant molecule of deteriorated seafoods, with high sensitivity and selectivity. However, the bioelectronic noses have not been so far compared with classical or conventional methods including sensory evaluation tests and chromatography analyses.

**Figure 1:**
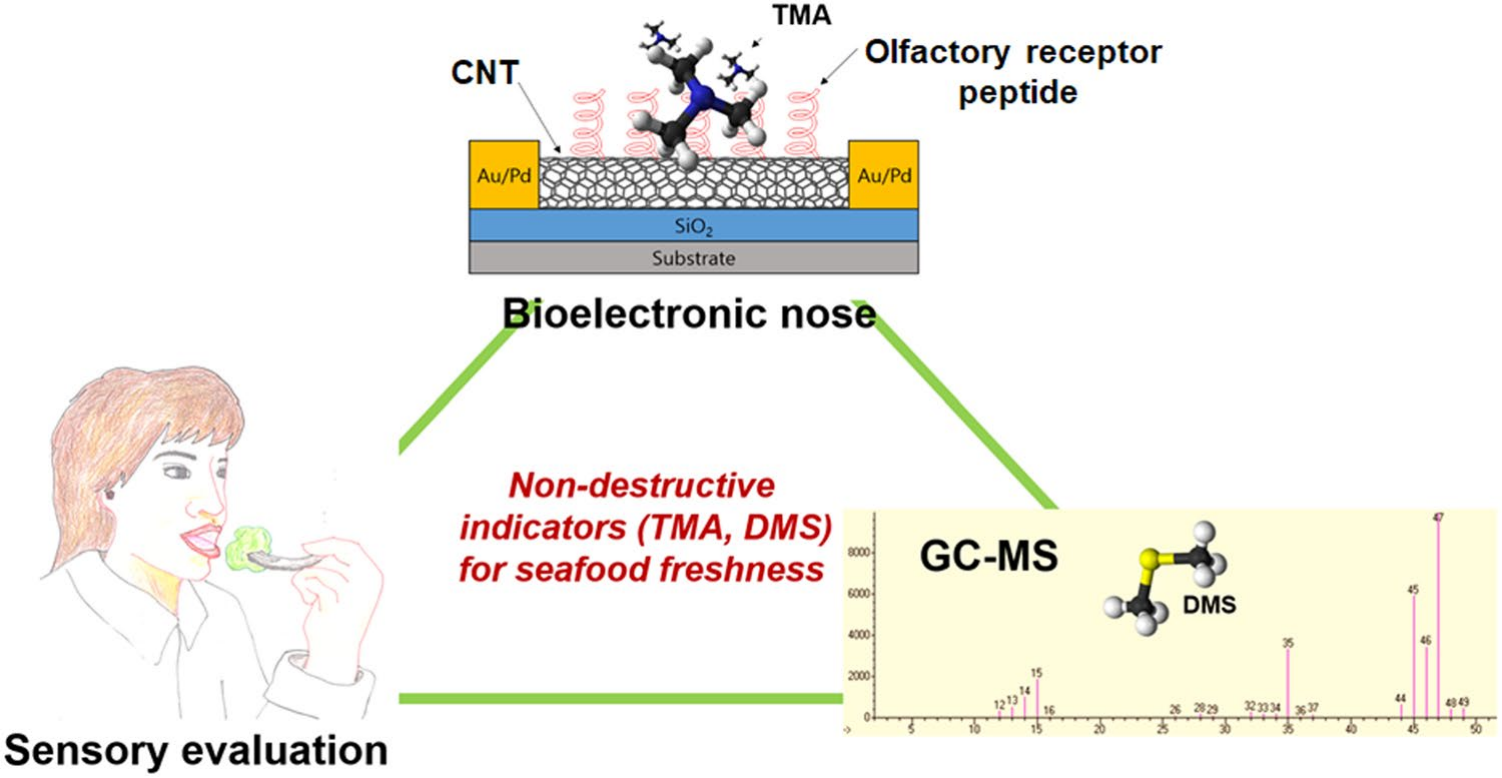
A triangle study of human evaluation test, instrumental analysis (GC-MS) and olfactory receptor/CNT bioelectronic nose for exploring non-destructive sensing of seafood freshness. The sensory evaluation test was performed by 15 trained panelists focusing on appearance, flavor, taste, texture and acceptability of raw oyster. For the instrumental analysis, the gas samples from the headspace of vials containing oysters were injected to the GC-MS system. Using the bioelectronic nose, a specific odorant molecule, TMA generated in the oyster undergoing the deterioration process was selectively detected through its binding to the receptor protein peptide.

In this study, we investigate a comparative triangle study combining sensory evaluation test by trained panellists, GC-MS analysis with or without SPME and the bioelectronic nose (Fig. 1) fabricated by incorporating human olfactory receptor peptide with carbon nanotube field-effect transistor (CNT-FET) to demonstrate an extraction-free, rapid and close-to-human monitoring of oyster freshness.

## Results and Discussion

### Sensory evaluation test of raw oyster

**Figure 2:**
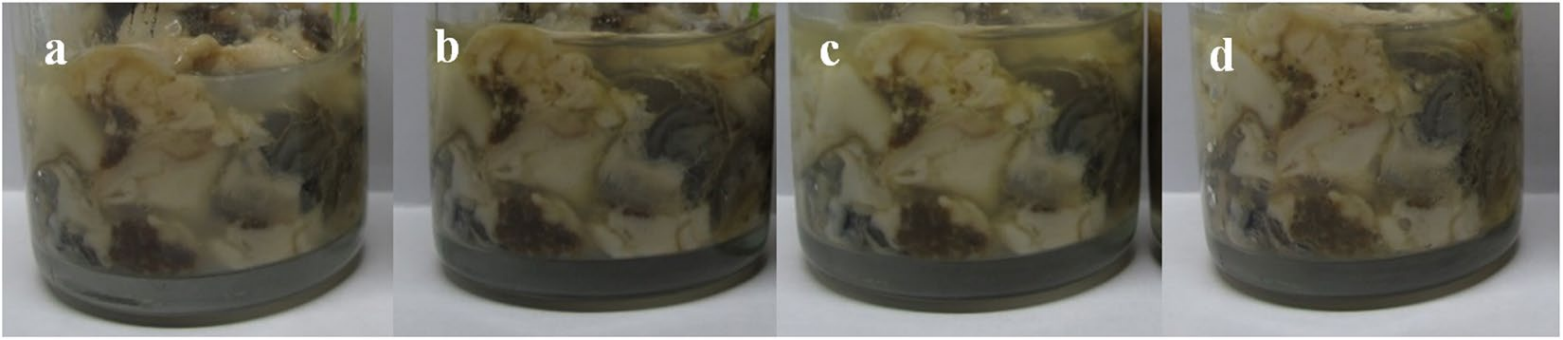
Appearance of raw oyster at 0 day (**a**), 2 days (**b**), 3 days (**c**) and 7 days (**d**) after storage at 4 °C, respectively. No dramatic changes of oyster appearance were found by naked eyes for 7 days post-harvest.

Sensory evaluation of raw oyster was performed by 15 trained panelists each day for 12 days after harvesting oysters with shell from a marine farm. The sensory evaluation index consisted of appearance, aroma, flavor, texture, taste, dislike to swallow, and overall acceptability (Table S1). The appearance of raw oyster (Fig. 2) gradually deteriorated over five or six days postharvest, resulting in damaged gills, mucilage, green spots, exudate water, and unclear transparency (Table 1). During the same period, the sensory scores for fresh and sea aroma/flavor decreased, whereas the score for a fish and pungent aroma/flavor increased, suggesting that the oyster underwent loss of freshness. The bitter and sour tastes implying the deterioration of oysters were also intensively sensed by a trained panel on these days; however, no dramatic change was found in the hardness. Overall, the acceptability of the raw oyster drastically decreased in six days post-harvest. However, the score for dislike to swallow was zero at five days post-harvest. The freshness of oysters by a sensory evaluation according to appearance, flavor, and taste was assessed as 50% of its original condition at 5 days post-harvest.

**Table 1:** Sensory evaluation of the oyster by 15 trained panelists. Fifteen trained panelists evaluated the freshness of raw oyster by scoring sensory characteristics including appearance, aroma/flavor, texture, taste, dislike to swallow, and overall acceptability. These characteristics were rated on a 10-point grading scale (0 = none; 10 = very intense) except dislike to swallow.

Index	Characteristics	Day 1	Day 2	Day 3	Day 4	Day 5	Day 6	Day 7	Day 8	Day 9	Day 10	Day 11	Day 12
Appearance	Gills	9.6 ± 0.6	8.1 ± 2.1	7.0 ± 2.2	6.1 ± 2.3	5.3 ± 1.9	5.4 ± 1.9	6.1 ± 2.2	4.6 ± 2.8	5.7 ± 2.5	5.4 ± 2.3	4.1 ± 1.9	4.8 ± 2.2
	Form of the Plum	9.8 ± 0.2	8.9 ± 1.5	7.9 ± 1.6	6.7 ± 1.6	5.4 ± 1.9	5.4 ± 2.0	5.7 ± 1.8	5.4 ± 1.9	5.8 ± 1.6	58 ± 1.8.	4.5 ± 1.4	5.0 ± 1.7
	Green spot	0.5 ± 1.3	1.3 ± 2.4	1.6 ± 2.0	1.6 ± 0.5	3.1 ± 2.2	3.9 ± 2.2	3.0 ± 2.1	3.7 ± 2.3	3.7 ± 2.2	3.7 ± 2.4	4.4 ± 2.6	5 ± 2.6
	Mucilage	1.8 ± 3.3	2.1 ± 2.3	3 ± 2.5	2.8 ± 2.5	3.4 ± 2.1	3.0 ± 1.8	2.7 ± 2.1	3.4 ± 1.9	3.6 ± 2.3	3.8 ± 2.0	3.4 ± 3.1	4.6 ± 2.5
	Exudate water	0.7 ± 1.1	2.2 ± 2.3	3.3 ± 2.4	5.4 ± 1.8	4.9 ± 1.6	4.5 ± 1.9	4.8 ± 1.7	4.6 ± 2.0	5.7 ± 2.4	5.6 ± 2.4	6.0 ± 2.6	6.9 ± 2.3
	Transparency	8.7 ± 2.2	6.2 ± 3.0	5.0 ± 2.7	5.5 ± 2.3	4.4 ± 2.0	4.0 ± 2.2	4.0 ± 2.4	3.0 ± 1.6	3.3 ± 2.0	3.2 ± 2.0	2.6 ± 1.7	2.5 ± 2.4
Aroma	Fresh	9.6 ± 0.7	8.3 ± 1.3	6.5 ± 1.7	5.6 ± 1.6	4.7 ± 1.6	3.7 ± 2.0	4.1 ± 1.6	4.0 ± 1.2	3.3 ± 1.2	3.3 ± 1.3	2.4 ± 1.2	2.6 ± 1.7
	Sea	9.0 ± 1.5	7.8 ± 1.7	5.6 ± 1.7	5.1 ± 2.2	4.5 ± 1.8	3.3 ± 1.7	4.1 ± 2.0	3.4 ± 1.4	3.5 ± 2.5	3.8 ± 1.7	2.6 ± 2.2	3.2 ± 2.4
	Fish & Pungent	1.5 ± 2.7	2.3 ± 2.1	2.7 ± 2.3	4.3 ± 2.4	5.6 ± 2.1	6.4 ± 1.5	6.6 ± 1.6	6.6 ± 1.5	6.5 ± 2.7	6.8 ± 1.8	8.0 ± 1.1	8 ± 1.7
Flavor	Fresh	9.7 ± 0.7	8.2 ± 1.4	6.5 ± 1.6	5.3 ± 1.8	5.6 ± 1.9	4.1 ± 1.6	4.1 ± 1.2	4.1 ± 1.2	3.9 ± 0.9	3.5 ± 1.0	2.5 ± 1.2	2.3 ± 1.9
	Sea	9.4 ± 1.3	7.0 ± 2.6	6.0 ± 1.6	5.0 ± 1.8	3.9 ± 2.0	3.9 ± 1.8	4.5 ± 1.7	3.1 ± 1.6	3.7 ± 1.1	3.0 ± 1.7	2.4 ± 1.7	2.9 ± 2.6
	Fish & Pungent	0.6 ± 1.0	2.0 ± 2.4	3.2 ± 1.9	4.3 ± 1.6	4.0 ± 2.3	6.1 ± 1.7	6.3 ± 1.7	5.4 ± 2.0	6.3 ± 1.9	6.4 ± 1.7	7.5 ± 1.9	7.9 ± 2.0
Texture	Hardness	6.5 ± 3.9	6.4 ± 2.9	5.4 ± 2.0	5.2 ± 1.8	4.2 ± 2.5	4.2 ± 1.7	4.8 ± 2.3	3.9 ± 2.3	4.3 ± 2.2	4.0 ± 1.9	3.0 ± 1.9.	3.5 ± 2.1
	Elasticity	9.1 ± 1.0	7.6 ± 1.8	5.6 ± 1.9	5.0 ± 1.9	5.5 ± 1.8	3.9 ± 1.6	3.7 ± 2.1	3.4 ± 2.0	3.1 ± 1.9	3.1 ± 1.8	2.4 ± 1.5	2.5 ± 2.1
Taste	Salty	7.3 ± 2.4	5.4 ± 2.9	4.7 ± 2.4	4.9 ± 2.1	5 ± 2.1	4.8 ± 1.8	4.9 ± 2.1	4.1 ± 1.7	4.71 ± 1.6	5.4 ± 2.1	3.8 ± 2.4	5.2 ± 2.6
	Bitter	0.1 ± 0.6	0.6 ± 0.9	2.2 ± 1.8	3.1 ± 2.2	2.9 ± 2.4	4.0 ± 2.8	3.8 ± 2.7	4.0 ± 2.7	4.2 ± 2.0	5.1 ± 2.1	6.3 ± 2.4	5.8 ± 2.6
	Sour	0.1 ± 0.2	0.5 ± 0.8	1.6 ± 1.3	2.6 ± 18	1.8 ± 1.2	3.3 ± 2.4	3.7 ± 2.8	3.2 ± 2.6	3.5 ± 2.1	4 ± 2.3	3.2 ± 2.3	3.8 ± 2.7
Acceptability	Overall-acceptability	9.6 ± 0.4	8.3 ± 1.0	6.6 ± 1.5	5.1 ± 1.4	4.4 ± 1.9	3.3 ± 1.4	3.3 ± 1.2	3.0 ± 1.6	2.9 ± 2.1	2.5 ± 1.5	1.6 ± 1.3	1.7 ± 2.1
	Dislike to swallow	0	0	0	0	0	7	9	9	10	10	11	12

### GC-MS detection of gaseous compounds from raw oyster

Based on the hypothesis that there are gaseous compounds vaporized in the earlier stage of food storage but hardly recognized by human sensory, we collected headspace gas samples from the vial containing raw oysters every day. Neither pre-treatment nor extraction for gas sampling was carried out for finding non-destructive indicator compounds. Headspace gas harvested in a syringe was injected into GC-MS to identify what compounds have been vaporized during the storage of raw oysters. At the GC-MS detectible concentration level (ppm), only one compound, dimethyl sulfide (DMS) was found and identified as an indicator of a food freshness change (Table 2 and Table S2) (Fig. S1). During the storage of oyster at 4 °C for 3 days post-harvest, DMS was not detected from the headspace gas in the vial containing raw oysters. However, the gaseous DMS started to be found in the headspace of raw oyster at 4 °C from 4 days post-harvest with concentration of 10.71 ± 0.12 ppm (Table 2) (Fig. S2b). The concentration of DMS in the headspace of oyster storage gradually increased until 9 days post-harvest (Table 2). Since the DMS is early detectible and continuously vaporized in the storage at 4 °C, the DMS is considered as a non-destructive marker for monitoring oyster freshness.

**Table 2:** Measurement of DMS from the oyster by GC-MS. The amount of DMS (ppm, w/w) was measured by the GC-MS analysis of gases in the headspace of vials containing oysters. All data were calculated using a calibration curve of standard DMS after the double measurements.

Temperature	Unit	Detection of dimethyl sulfide (DMS) from the headspace of oyster storage								
		Day 1	Day 2	Day 3	Day 4	Day 5	Day 6	Day 7	Day 8	Day 9
4 °C	ppm (S.D.)	—	—	—	10.71 (0.12)	11.69 (0.30)	12.73 (0.05)	12.93 (0.08)	13.80 (0.70)	15.51 (0.50)
36 °C	ppm (S.D.)	100.05 (15.25)	165.82 (30.05)	275.10 (20.13)	348.35 (16.80)					

^*^S.D. Standard Deviation.

In case of storing raw oysters at 36 °C, we detected DMS from the headspace at 1 day post-harvest (Fig. S2d) with high concentration (100.05 ± 15.25 ppm), which was 3 days earlier than the storage at 4 °C. Since the deterioration of raw oyster freshness progressed faster at the higher temperature, the speed and intensity of DMS generation increased at 36 °C. A strong dependence of temperature implies that a bacterial contamination led to the food spoilage with generating DMS in raw oysters. The odor threshold of DMS is less than 1 ppm (Fenaroli’s Handbook of Flavor Ingredients); however, neither radish nor cabbage odor was recognized from the oyster at 4 days post-harvest (4 °C). As shown in the sensory evaluation test, less than 50% panellists were able to smell Fish & Pungent aroma or flavor from the oyster sample at 4 days post-harvest (Table 1).

SPME (Solid Phase Micro Extraction) is known as an efficient method to harvest gaseous molecules. Since gas compounds are continuously absorbed and concentrated on a fiber, multiple unknown organic molecules even with a small concentration are detectible and analysable using GC-MS. To identify other gaseous compounds except DMS, headspace gases vaporized from raw oyster were collected onto the SPME fiber and were consequently analyzed by GC-MS (Fig. S3). Butanoic acid and 4-methyl-pentanoic acid were particularly detected in the headspace of raw oysters from the first day of incubation at 36 °C (Table 3). DMS was not found by SPME-GC-MS because this fiber is optimized for harvesting organic compounds containing more than three carbons (≥ C_3_). In case of storing oyster at 4 °C, none of GC peaks for headspace gas compound was captured by SPME-GC-MS, which indicates that those organic components including butanoic acid and 4-methyl-pentanoic acid were not favourably generated at a lower temperature. These results correlate with the data from sensory evaluation test because the panel did not recognize the odor of these organic compounds. Moreover, the result demonstrate that microorganisms exist in the oyster; however, their activities were strongly suppressed by the low temperature.

**Table 3:** Bacterial species producing DMS and TMA were found and identified from the raw oysters with losing their freshness. The 16S rRNA genome of complex microbiomes in raw oyster used in this study was determined by the duplicate samples prepared from the soup of oyster at 4 days post-harvest. Among the total reads (248,615), 94.67% and 72.89% were classified to genus and species level, respectively.

Genus	Species	Genus	Species
*Flavobacterium*	*succinicans branchiophilum*	*Halomonas*	*almeriensis*
*Pseudoalteromonas*	*aliena gracilis issachenkonii haloplanktis denitrificans*	*Pseudomonas*	*clemancea azotoformans*
*Shewanella*	*sediminis pneumatophori piezotolerans livingstonensis baltica*	*Vibrio*	*litoralis neonatus chagasii gallaecicus bacterium*

### Fabrication and application of bioelectronic noses for the monitoring of raw oyster freshness

**Figure 3:**
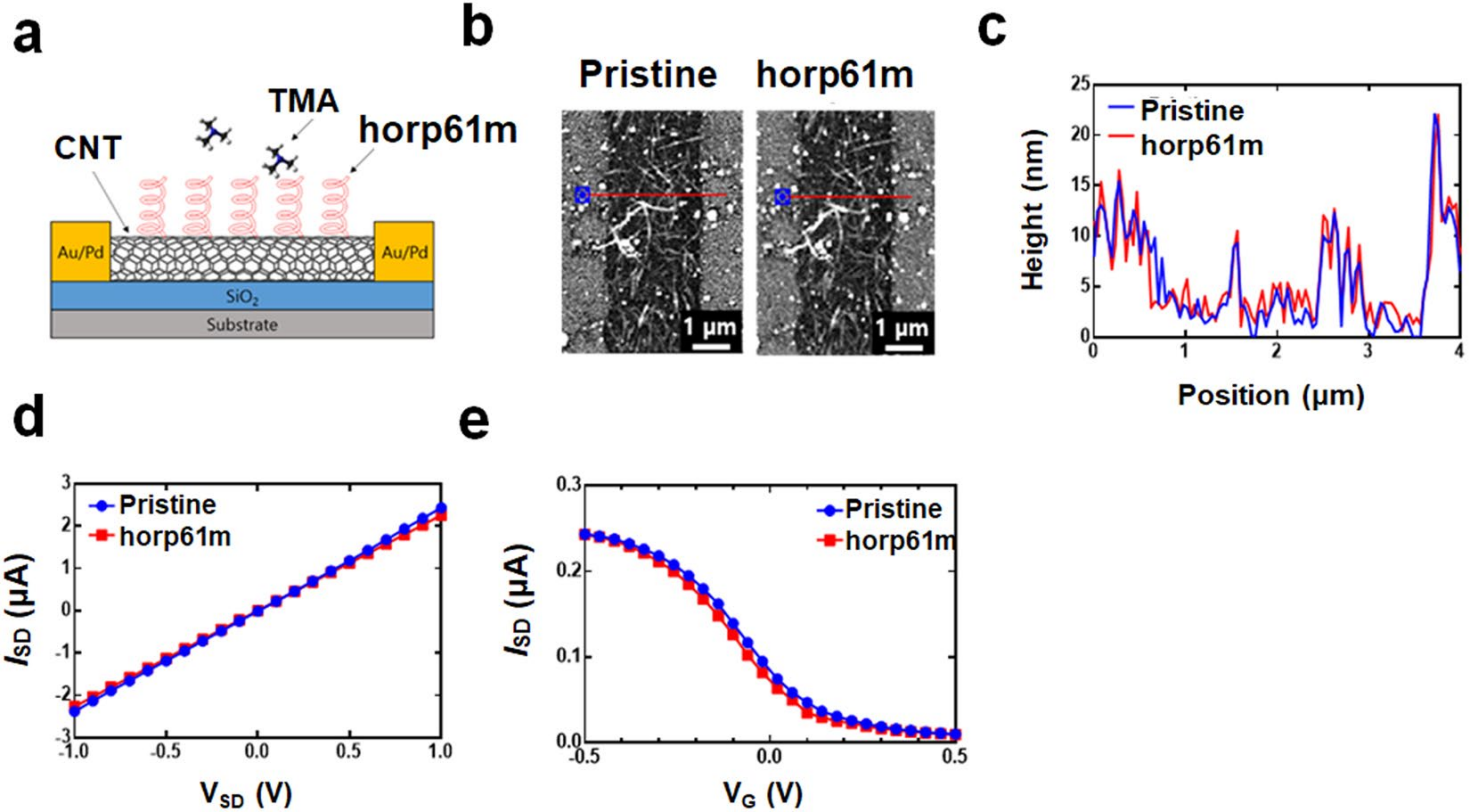
Fabrication of a peptide-based bioelectronic nose. (**a**) Schematic diagram of the bioelectronic nose. AFM images (**b**) and height profiles (**c**) of pristine and the olfactory receptor peptide coated CNT channels. Current-voltage characteristics (**d**) and gate profiles (**e**) of CNT-FET before and after the immobilization of the peptide.

Trimethylamine (TMA) is an odorant compound generated in seafood undergoing spoilage by bacterial contamination. In the previous study, a bioelectronic nose using human olfactory receptor peptide could detect TMA from spoiled seafood^21^. To evaluate whether TMA detection might determine raw oyster freshness, we fabricated carbon nanotube (CNT)-based bioelectronic noses. For the selective recognition of TMA, the peptide with three additional molecules of phenylalanine (Phe) at C-terminus was synthesized^22,23^. The synthesized peptide, horp61m with a function of TMA binding was specifically immobilized onto CNTs via pi-pi interactions of aromatic rings^23,24^ (Fig. 3a). The atomic force microscopy (AFM) images of the CNT channel before and after the functionalization of the horp61m were analyzed to confirm the immobilization of the peptide (Fig. 3b). The height of the CNT channel was increased by 1.96 ± 0.28 nm after the immobilization of the peptide (Fig. 3c). To evaluate the electrical property of the peptide-functionalized CNT-FET, the current-voltage characteristics of source-drain (*I*_SD_-*V*_SD_) were measured (Fig. 3d). The resistance increased from 4.01 × 10^5^ Ω to 4.22 × 10^5^ Ω, and the linearity of the curve was maintained after the immobilization of the peptide. The current between the source and the drain (*I*_SD_) decreased with an increasing liquid gate voltage (*V*_G_) (Fig. 3e); however, a p-type FET characteristic was retained in the sensor. These results indicate that the peptide was successfully immobilized onto the CNT channel with resulting in the detection of positively-charged TMA molecules, an indicator of seafood freshness^16,25^.

**Figure 4:**
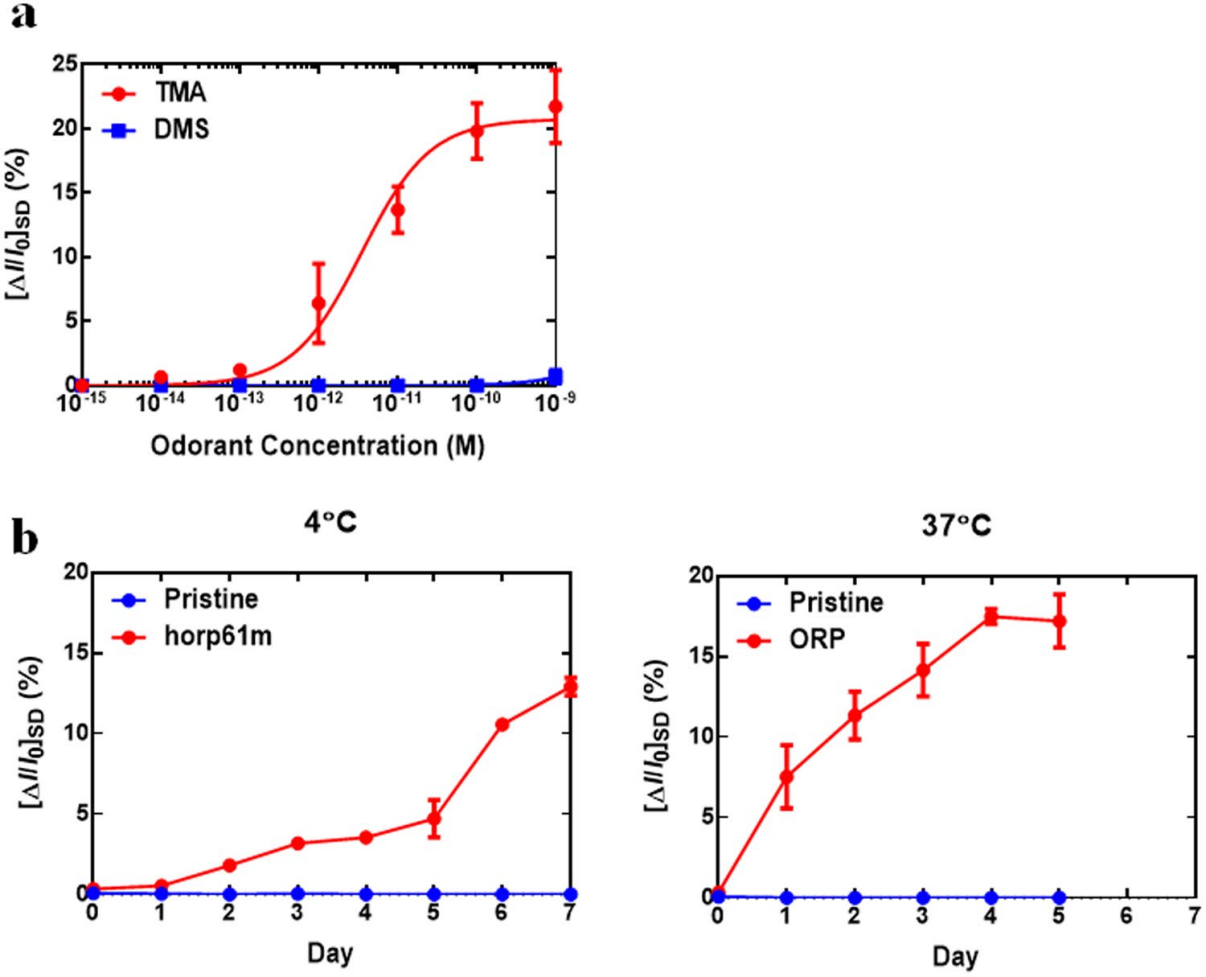
Detection of TMA and oyster deterioration by the bioelectronic nose. (**a**) Dose-dependent electric signal responses of the bioelectronic nose to TMA and DMS. (**b**) Responses of the bioelectronic nose to raw oyster samples stored at 4 °C for 7 days and 37 °C for 5 days, respectively.

Figure 4a shows the conductance changes of the bioelectronic nose with the concentrations of TMA ranging from 1 fM to 1 µM. The bioelectronic nose sensor selectively responded to TMA, whereas no signal was found in the response to DMS. As reported in the previous study, the molecules with similar molecular sizes or chemical structures to TMA did not bind with the receptor protein or peptide^21^. Therefore, the bioelectronic nose sensed only TMA though the oyster generated and contained large amounts of DMS along with TMA from 4 days post-harvest. The detection limit of the bioelectronic nose was 10 fM, and the relative standard deviation (RSD) value was 34.37% with 10 fM of TMA. The equilibrium constant *K*_d_ was estimated as 3.33 × 10^12^ M^−1^ using the Langmuir isotherm model. To evaluate the sensing ability of the bioelectronic nose for the assessment of seafood quality, we used oyster samples stored at 4 °C for 0–7 days and 37 °C for 0–5 days, respectively (Fig. 4b). The oyster samples were diluted by 1:100 in distilled water to avoid a non-specific response. The pristine CNT-FET did not respond to oyster samples, but the current signals were changed when oyster samples were injected to the horp61m-functionalized CNT-FET. These results indicate that compounds produced from spoiled oysters could not affect the conductance of the pristine CNT-FETs, and the binding events of the olfactory receptor-derived peptide with TMA on the CNT surface selectively affected that of the horp61m-functionalized CNT-FETs. The conductance of the sensors was changed by the injection of sample solutions stored at 4 °C for 2 days and those stored at 37 °C for 1 day (Fig. 4c). We observed the enhanced sensor signals when long term-stored oyster samples were injected. The larger sensor signals were observed with the higher temperature where seafood spoiled rapidly. These were similar to the results observed in GC-MS methods, while the sensor began to respond to the samples stored at a room temperature for two-days shorter than the GC-MS method.

The most attractive advantage of bioelectronic nose is its sensitivity to the target non-destructive odorant indicator, TMA. Both GC-MS and SPME-GC-MS did not detect TMA from the same amount and batch of oyster that was used in the bioelectronic nose assessment for the same period (0 ~ 7 days) of storage at 4 °C. The reason for a difficulty in the TMA detection by GC-MS was because the concentration of TMA in the headspace of oyster was too low to be detected by GC-MS. When the standard molecule of TMA was analyzed by GC using serial dilutions, more than 0.1% (v/v) TMA could be found in GC analysis (Fig. S4). These results indicate that the TMA was included in the headspace of oyster with a concentration less than 0.1%, suggesting no signal in GC histogram. However, the bioelectronic nose is so sensitive (10^−13^ M, Fig. 4a) that the TMA could be detected by this olfactory sensor in the earlier storage time (2 days post-harvest, Fig. 4b).

### Interactive relevance among the sensory evaluation, GC-MS and bioelectronic nose

During the storage of oyster at 4 °C, TMA was first detected by the bioelectronic nose at 2 days post-harvest, and DMS was first detected by GC-MS at 4 days post-harvest, respectively. However, more than 50% of the trained panel evaluated the oysters as acceptable to swallow with intact appearance and odors. Given that the freshness of raw oyster is defined as a short interval of storage time after harvest, the detection of TMA and DMS constitute a promising method of food freshness assessment because their concentrations increased in proportion to the time particularly in the earlier period of storage.

**Figure 5:**
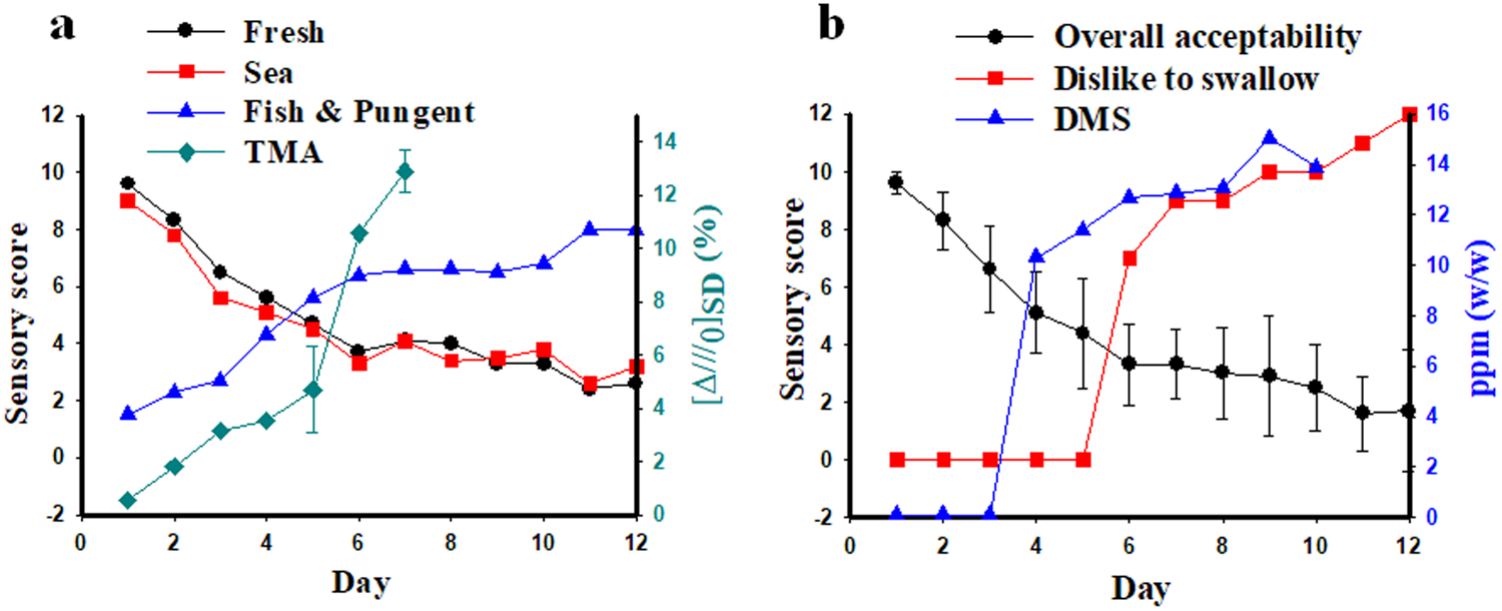
Interactive relevance among sensory evaluation, GC-MS, and bioelectronic nose. (**a**) Correlation profile between flavor index and TMA detection by the bioelectronic nose. (**b**) Correlation profile between overall acceptability and DMS detection by GC-MS.

Because TMA has a characteristic fishy and ammonia-like odor, its detection profile by the bioelectronic nose was investigated by coupling with a fish and pungent aroma/flavor profile by sensory evaluations (Fig. 5a). The fish and pungent aroma/flavor score evaluated by the trained panel increased with a similar profile of TMA generation detected by a bioelectronic nose. The result implies that TMA from oysters losing its freshness contributed to the increase in fish and pungent aroma/flavor, as well as to the reduction of fresh aroma/flavor in the sensory evaluation test. DMS has a cabbage and sulfurous odor in a certain concentration, which led people to feel particularly unpleasant about food. When the time-course DMS generation profile was compared with sensory evaluation score, dislike to swallow showed an increasing pattern similar to the DMS generation (Fig. 5b). Although the score for overall acceptability gradually decreased over the storage period, the trained panel did not feel a dislike to swallow until after five days of storage. Interestingly, DMS was detected by GC-MS at 2 days before dislike to swallow started to be scored by the trained panel.

Most of the sensory evaluation index was rapidly increased after the start of raw oyster storage in a refrigerator at 4 °C (Table 1). TMA and DMS were not detected after 1 day of storage, while some of the sensory evaluation index showed a defect in freshness including its appearance and texture. Although the trained panel could recognize a degree of damage and defective shape in raw oysters, it was generally difficult to tell a fresh oyster from a defective one. The overall appearance of raw oyster in a glass vial looked similar despite longer storage times of 2, 3 and 7 days (Fig. 2). Without professional experience of observing fresh raw oyster, any reduction in freshness was too subtle to detect just by observing its appearance.

Bioelectronic noses recognizes a single odor when only one olfactory receptor protein or peptide is used for the CNT sensor. However, multiple odors of oysters were simultaneously sensed by panel’s nose upon conducting sensory evaluation. In addition to such diversity of flavors from food, environmental conditions and circumstances may interfere with panel’s determination to evaluate oyster freshness. Bioelectronic noses with double recognition system using olfactory receptors for both TMA and DMS would be useful to monitor oyster and seafood freshness because most of the seafood generates these odorant molecules through enzymatic reactions. Bacterial cells that inhabit oysters or its food corals contain such enzymes to produce TMA and DMS.

### Identification of bacterial contamination in raw oyster

**Figure 6:**
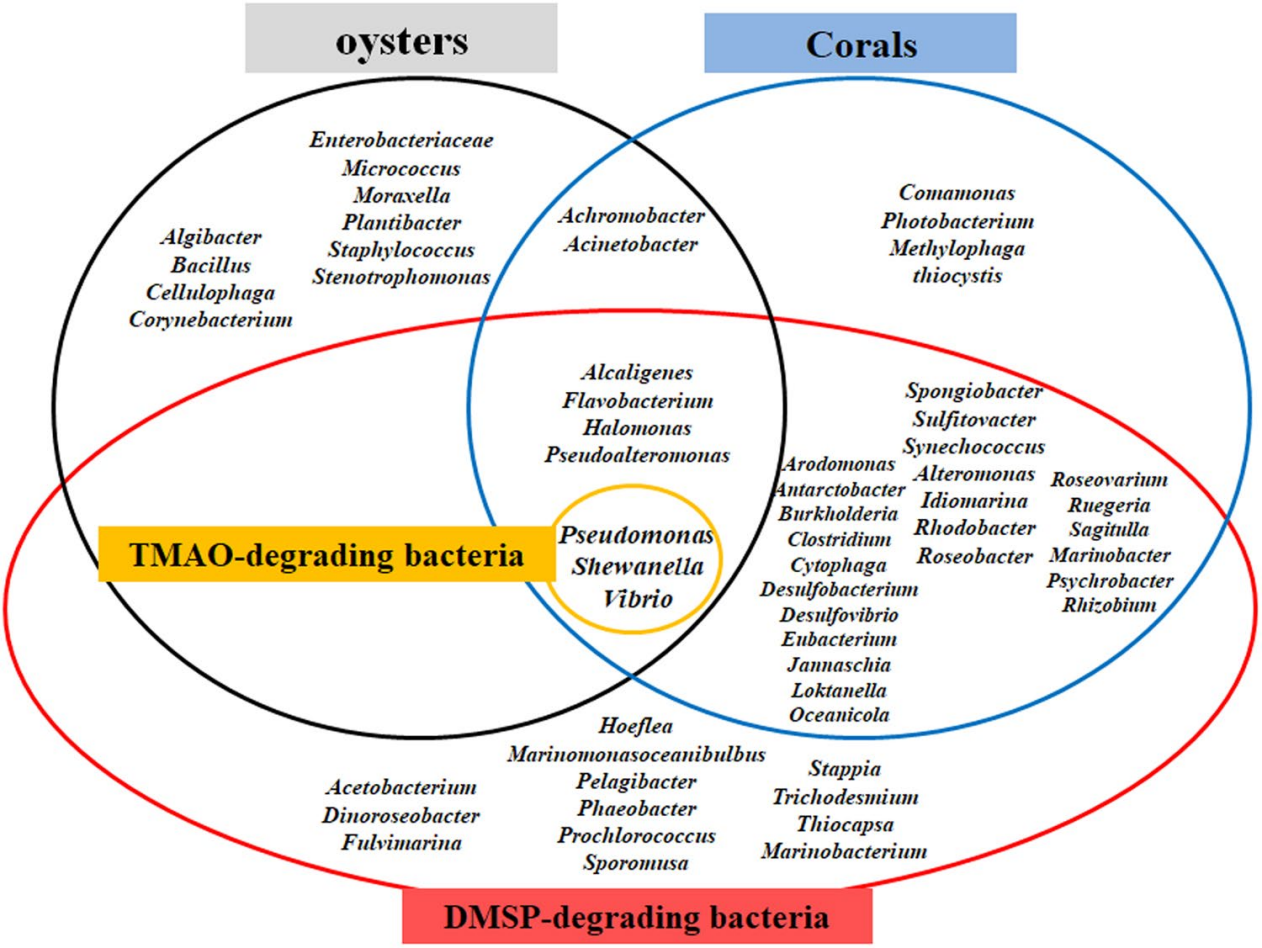
Bacterial taxonomy in raw oyster. A Venn diagram indicates groups of bacteria living in oyster or corals producing TMA or DMS. TMA and DMS are produced in each bacterial group through the degradation of TMAO and DMSP, respectively.

Among the bacterial taxa that oyster and corals harbor, some species are known to convert trimethylamine N-oxide (TMAO) and dimethylsulfoniopropionate (DMSP) into respectively TMA and DMS through an enzymatic reaction^26–31^. *Pseudomonas*, *Shewanella*, and *Vibrio* are representative bacterial genera found in oyster and are known to produce TMA (Fig. 6). In addition, *Alcaligenes*, *Flavobacterium*, *Halomonas*, and *Pseudoalteromonas* are harbored in oyster and are known to generate DMS. The growth of such bacteria in raw oysters leads to the reduction in freshness through deteriorating food qualities including flavor, taste and texture. Some of these species threaten food safety by triggering an outbreak of food poisoning through the production of harmful toxins.

To investigate whether bacterial contamination caused the generation of TMA or DMS, we identified bacterial taxa in the raw oysters using 16 S rRNA metagenomics (Illumina, Inc.). Some species of *Flavobacterium*, *Halomonas* and *Pseudoalteromonas* were identified from the raw oysters used in this study (Table 3). These are known to produce DMS by consuming DMSP in oysters and corals. Some species of *Pseudomonas*, *Shewanella*, and *Vibrio* were also identified from the oyster used in this study. These bacteria are known to inhabit oysters and produce TMA through the consumption of TMAO in oysters or corals as well as DMS from DMSP. Given that some of these species are involved in gastrointestinal illnesses, the rapid and early detection of TMA or DMS using bioelectronic noses is helpful to prevent foodborne diseases or outbreaks. Given that identification of bacterial species and counting their colonies are thought to be time- and labor-intensive procedure, DMS detection by GC-MS is also fast and non-destructive method to recognize bacterial contamination of seafoods because those bacterial species producing DMS rapidly deteriorate food safety and quality.

Given that the deterioration of food is judged and featured by the amount of toxins or harmful microorganisms in the food, the detection of TMA or DMS from oysters by bioelectronic nose or GC-MS does not directly mean that the oyster should be immediately discarded for the safety. The quantitative detection of these non-destructive markers indicates a change in oyster freshness rather than a discrimination between good and bad. Because some people with the weaker immune system are sensitive to a small amount of toxin or harmful microorganism in foods, the rapid and quantitative monitoring of freshness by detecting these markers is very useful for the prevention of bowel illnesses and food poison outbreaks. In addition, the information of those non-destructive markers can be used for the automatic and systematic grading of seafood quality without panel’s sensory evaluation test since the freshness corresponds to the quality of seafood. In this study, the trained panelists evaluated the oyster as an acceptable, whereas sensor and instrumental analysis recognized the freshness change by detecting TMA and DMS in the early phage of food storage. During the late phage of post-harvest storage, along with TMA or DMS detection by bioelectronic nose and GC-MS, the panelists recognized the reduction of food quality or freshness from these oysters with result in lower scores of the sensory evaluation. Based on these results, the present study contributes to the disclosure of relationship between freshness and deterioration.

To materialize real-time and on-site monitoring of food freshness or deterioration, the finding of non-destructive marker (a target compound) is an essential prerequisite. Gaseous compounds generating from foods in the storage condition are the most useful non-destructive markers because the sampling procedures or steps can be diminimized or omitted in the monitoring process. Since those gaseous compounds are quickly diffused and mixed with other gaseous molecules, a sensor with high sensitivity and selectivity is required for the use of these non-destructive gaseous markers. The bioelectronic nose has a great potential for sensing the non-destructive markers due to its fM scale sensitivity and olfactory receptor-based selectivity. Therefore, the finding of specific odorant molecule such as TMA that indicates the reduction of food freshness or the progress of food deterioration is necessary for the use of bioelectronic nose. If there are gaseous compounds extensively and early generated during the food deterioration process, those molecules can be the non-destructive markers to be used for GC-MS instrumental analysis. In this context, DMS is another strong candidate of non-destructive marker for seafood freshness monitoring using GC-MS.

## Conclusions

By the comparative triangular study using sensory evaluation test, GC-MS instrumental analysis and bioelectronic nose sensing, we suggest the non-destructive markers, DMS and TMA, to quantitatively determine raw oyster freshness and to early monitor its post-harvest deterioration process. DMS and TMA were simply detected by GC-MS and bioelectronic noses, respectively, without time- and labor-consuming destructive food preparation processes. The sensory evaluation test revealed that the trained panelists started to recognize fish/pungent odors and changes in texture of oyster from 1 day post-harvest. However, all the panelists did not feel the dislike to swallow of oyster until 5 days post-harvest. The bioelectronic nose was fabricated by immobilizing the peptides of human olfactory receptor protein onto CNTs, which enables a binding of TMA from the oyster with the peptides to generate a current change with high sensitivity (fM scale). GC-MS analysis showed a deterioration of oyster from 4 days post-harvest using DMS as an indicator (ppm scale), whereas the bioelectronic nose recognized the reduction of oyster freshness from 2 days post-harvest by sensing TMA. The bacterial taxa analysis was performed and suggested that *Pseudomonas*, *Shewanella*, and *Vibrio* producing both TMA and DMS were contaminated in the raw oyster. Given that some of bacterial species producing DMS or TMA along with food poisoning toxins were found in the oyster, a bacterial contamination-driven food deterioration is rapidly detected using the bioelectronic nose. These non-destructive markers, DMS and TMA have a great potential for the real-time and on-site seafood freshness monitoring to prevent biological contamination-induced foodborne illnesses and food poison outbreaks.

## Materials and Methods

### Raw oyster

Raw oysters, *Crassostrea gigas* in shell were used for comparative analysis. Oysters were purchased from fisher market located on the Yellow Sea, Korea on the same day as harvest. Oyster samples for assessment were prepared without any washing or pretreatment and separated for three groups: sensory evaulation test, GC-MS analysis and bioelectronic nose sensor.

### Sensory evaluation test

Sensory evaluation was performed on the oysters stored for 12 days at 4 °C after harvest. Oyster terms were selected that broadly represented raw oyster quality through three rounds of training with fresh oysters (reference) and oysters stored for 10 days. Panelists were required to score the intensity of each characteristic describing appearance (shape of gills, form of the plum, green spots, mucilage, exudate water, transparency), aroma/flavor (fresh, sea, fish and pungent), texture (hardness, elasticity), taste (salty, bitter, sour), dislike to swallow, and overall acceptability. These sensory characteristics were evaluated on a 10-point grading scale (0 = none; 10 = very intense) except dislike to swallow (Table S1) throughout the study. Sensory sessions were organized throughout the storage period (12 days), and sampling of oysters occurred 1 h before the test. Each panelist received three oysters taken from the refrigerator at 4 °C. The Statistical Analysis System (SAS 9.1) program was used for statistical analysis of the sensory evaluation. Sensory evaluation was performed in Chung-Ang University where the IRB (institutional review board) approval was not necessary for the sensory evaluation test for foods at that time, 2014. The panelists were recruited by obtaining the fully informed consent from them after explaining all experimental protocols and methods.

### GC-MS for the identification of gaseous compounds

Each 50 g of raw oyster was stored in 100 mL glass vials sealed with rubber septum cap at 4 °C and 36 °C for 10 days, respectively. To prepare GC-MS samples vaporized from oyster, gaseous compounds in the headspace of vial were obtained on each day using a syringe or SPME fiber (50/30 μm StableFlex Divinylbenzene/Carboxen^TM^/Polydimethyl-siloxane, Supelco, Belleonate, PA, USA). To avoid mixing of gases between inside and outside of oyster vials, a needle with syringe was injected through the rubber of septum cap, and 1 mL of gas was collected into the cylinder of syringe. Gas samples were injected into GC instrument (Agilent 6890, Agilent Technologies, USA) coupled with a mass selective detector (Agilent 5973). Compounds were separated in a capillary column (HP-5, 30.0 m length x 0.25 mm I.D. x 0.25 μm film thickness). Temperature of the column was initially maintained at 40 °C for 3 min and increased up to 130 °C (15 °C/min). The column was consequently heated up to 280 °C (10 °C/min) and finally kept for 5 min. Injector temperature was set at 250 °C with a 1:10 spilt ratio. Helium was employed as carrier gas with a flow rate of 1.0 ml/min. The mass spectrometer was operated in the electron ionization mode (1,400 eV) with ion source temperature at 230 °C. Chemical compounds were detected in the scan mode with a mass range of m/z 20–500 amu and identified using NIST (National Institute of Standards and Technology) mass spectrum database.

### Fabrication and electrical measurement of bioelectronic nose

The construction process of the bioelectronic nose was similar to that in our previous reports^21^. In brief, octadecyltrichlorosilane (OTS) was patterned on a silicon oxide (SiO_2_) wafer via the photolithography method. The OTS-patterned wafer was dipped into swCNT solution (0.05 mg/mL in dichlorobenzene) for 20 s. CNTs were specifically absorbed on the bare SiO_2_ regions. Afterward, Ti (10 nm)/Au (30 nm) electrodes were developed via thermal evaporation and covered with a photoresist layer (DNR) to prevent the electrodes from contacting the solution. Human olfactory receptor-derived peptide (horp61m: NQLSNLSFSDLFFF) with purity higher than 95% was purchased from Peptron (South Korea). The peptide was suspended at 1 mg/mL with distilled water. For the immobilization of the peptide, 1 µL of peptide solution was placed on the CNT channel for 4 h at a room temperature. After the immobilization process, the CNT channel was washed with distilled water for 3 times to remove unbound peptides (Fig. S5). The image of the CNT channels was acquired before and after the immobilization of the peptide using AFM (Asylum Research, USA) in tapping mode with a scan rate of 0.2 Hz. For the electrical signal measurement, 49.5 µL of distilled water droplet was placed to the CNT channel, and 0.5 µL of the sample solution with the odorant and the oyster was injected. A bias voltage of 0.1 V was applied between the source and the drain, whereas the gate voltage was grounded. The source-drain current was monitored with a 2636 A Dual-channel system source meter instrument (Keithley, USA) and a MST 8000 probestation (MS TECH., South Korea).

### Identification of bacteria from complex microbiomes in raw oyster

The 16S rRNA genome sequencing of complex microbiomes in raw oyster used in this study was performed by the cooperation with the expertized company, LAS (South Korea) using the illumina 16 S metagenomics system (HiSeq. 2000, USA). Duplicate samples were prepared from the soup of oyster at 4 days post-harvest and sent to LAS at the same day. Among the total reads (248,615), 94.67% and 72.89% were classified to genus and species level, respectively. According to the classification results by taxonomic level, 99.93% were bacteria, and 0.07% were unclassified at kingdom level.

## Electronic supplementary material

Supplementary Information
