# Supplementary material for: A triangle study of human, instrument and bioelectronic nose for non-destructive sensing of seafood freshness
Source: Sci Rep. 2018 Jan 11;8:547. doi: 10.1038/s41598-017-19033-y (PMC5765080; doi:10.1038/s41598-017-19033-y)
Supplement: Supplementary file 1 — Supplementary Information [file 41598_2017_19033_MOESM1_ESM.pdf]

Supplementary Information

A triangle study of human, instrument and bioelectronic nose for non-destructive sensing of seafood freshness

Kyung Mi Lee[1,2,†], Manki Son[3,†], Ju Hee Kang[4], Daesan Kim[5], Seunghun Hong[5,6], Tai Hyun Park[3,7,8], Hyang Sook Chun[4], Shin Sik Choi[1,2]*

[1] Department of Food and Nutrition, Myongji University, Yongin, Gyeonggi 449-728, Republic of Korea
[2] Department of Energy Science and Technology, Myongji University, Gyeonggi 449-728, Republic of Korea
[3] Interdisciplinary Program for Bioengineering, Seoul National University, Seoul 151-742, Republic of Korea
[4] Department of Food Science and Technology, Chung-Ang University, Ansung, Kyonggi 456-756, Republic of Korea.
[5] Department of Biophysics and Chemical Biology, Seoul National University, Seoul 151-747, Republic of Korea
[6] Department of Physics and Astronomy and Institute of Applied Physics, Seoul National University, Seoul 151-742, Republic of Korea
[7] School of Chemical and Biological Engineering, Seoul National University, Seoul, Republic of Korea
[8] Advanced Institutes of Convergence Technology, Suwon 433-270, Republic of Korea

[†]These authors contributed equally to this work.
*Correspondence and requests for materials should be addressed to S.S.C. (email: sschoi@mju.ac.kr)

Table S1. Sensory evaluation parameters and scales for score.

	Sensory parameters	Scales for score 0	10
Appearance	Gills, Form of the plum	Defective form	Intact form
	Green spot, Mucilage, Exudate water	Absent	Very intense
	Transparency	Unclear	Clear
Aroma	Fresh, Sea, Fish & pungent	Absent	Very intense
Flavor	Fresh, Sea, Fish & pungent	Absent	Very intense
Texture	Hardness	Very soft	Very hard
	Elasticity	Absent	Very elastic
Taste	Salty, Bitter, Sour	Absent	Very intense
Dislike to swallow	-	Number of panel	
Overall acceptability	-	Dislike extremely	Like extremely

Table S2. Detection of DMS from the oyster harvested in different area and stored at 4°C or 36°C using GC-MS.

Temperature	Oyster	Unit	Amount of dimethyl sulfide (DMS)		
			Day 1	Day 2	Day 3
36°C	25 g	% of peak area (ppm)	6.67 (0.86)	6.27 (0.81)	6.85 (0.88)
	50 g	% of peak area (ppm)	10.39 (1.34)	10.09 (1.30)	10.40 (1.34)
4°C	1 g	% of peak area (ppm)			30.31 (0.32)

Table S3. Measurement of organic compounds vaporized from oyster by SPME and GC-MS.

Peak No.	RT (min)	Compound	% of peak area					
			Control	Day 1	Day 3	Day 4	Day 5	Day 7
a	5.13	Butanoic acid	-	41.55	37.48	34.13	29.76	23.99
b	8.08	4-methyl-pentanoic acid	-	25.12	13.43	16.95	13.66	4.11
c	10.08	2-nonanone	-	-	-	2.38	2.22	4.21
d	11.29	4-ethyl-benzaldehyde	-	-	-	-	1.90	4.30
e	11.93	Decanal	-	-	-	-	0.74	1.08
f	12.88	(E)-2-decenal	-	-	-	-	1.08	1.55
g	13.22	2-undecanone	-	-	-	-	1.86	2.83
h	13.88	3-undecen-2-one	-	-	-	1.38	1.84	3.57
i	18.25	Heptadecane	-	-	-	-	0.74	0.83

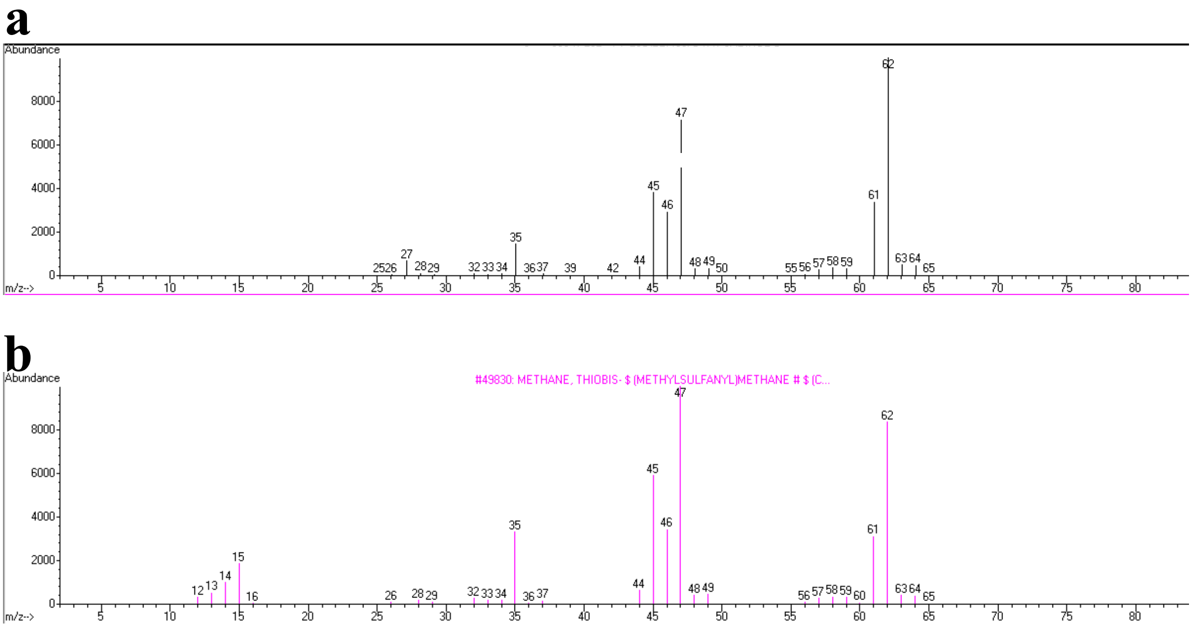

Figure S1. Identification of headspace gaseous samples vaporized from raw oyster using GC-MS. (a) Chromatogram of compound found in gaseous sample vaporized from oyster during the storage at 4°C for 4 days. (b) Mass spectrum of DMS in the database of NIST library.

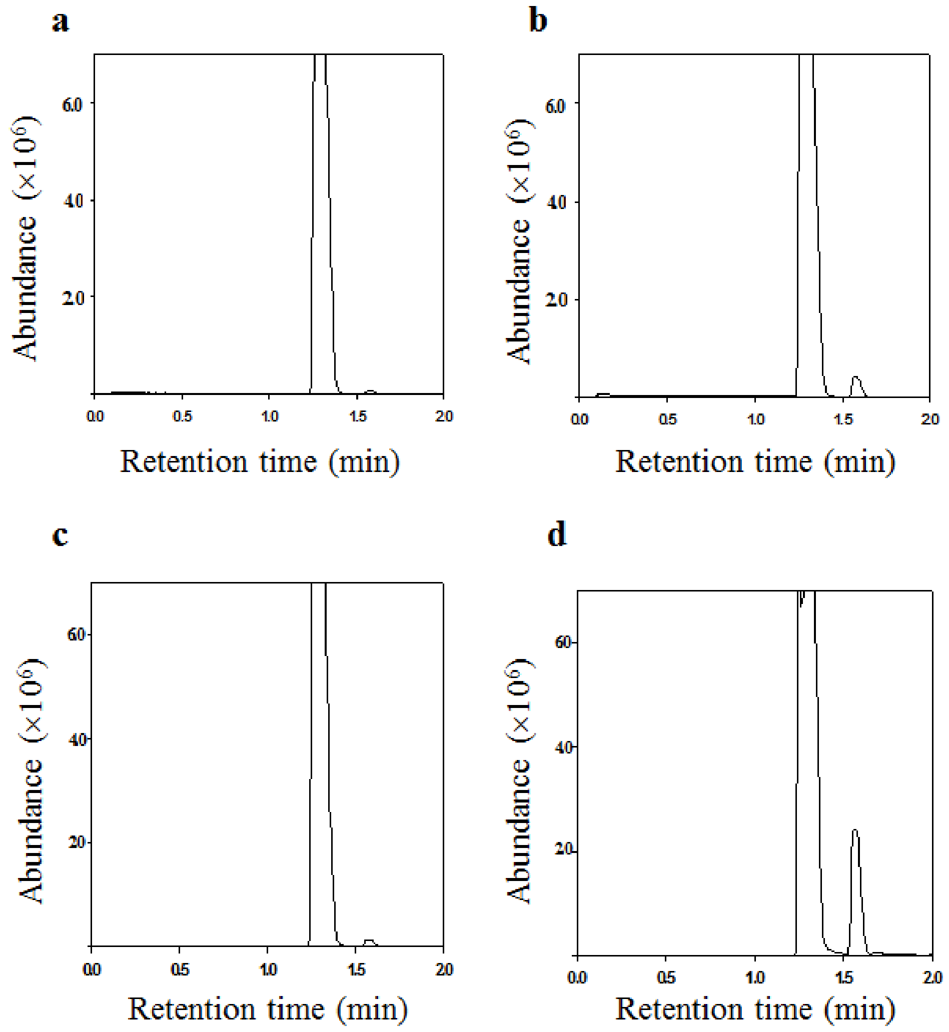

Figure S2. Detection and identification of gaseous markers indicating deterioration of oyster freshness. The DMS GC peak was not found at 0 day (a) post-harvest but first detected from the headspace of the oyster at 4 days (b) after storage of raw oyster at 4°C. In case of storage at 36°C, the DMS peak was not found at 0 day (c) but first detected at 1 day (d) post-harvest.

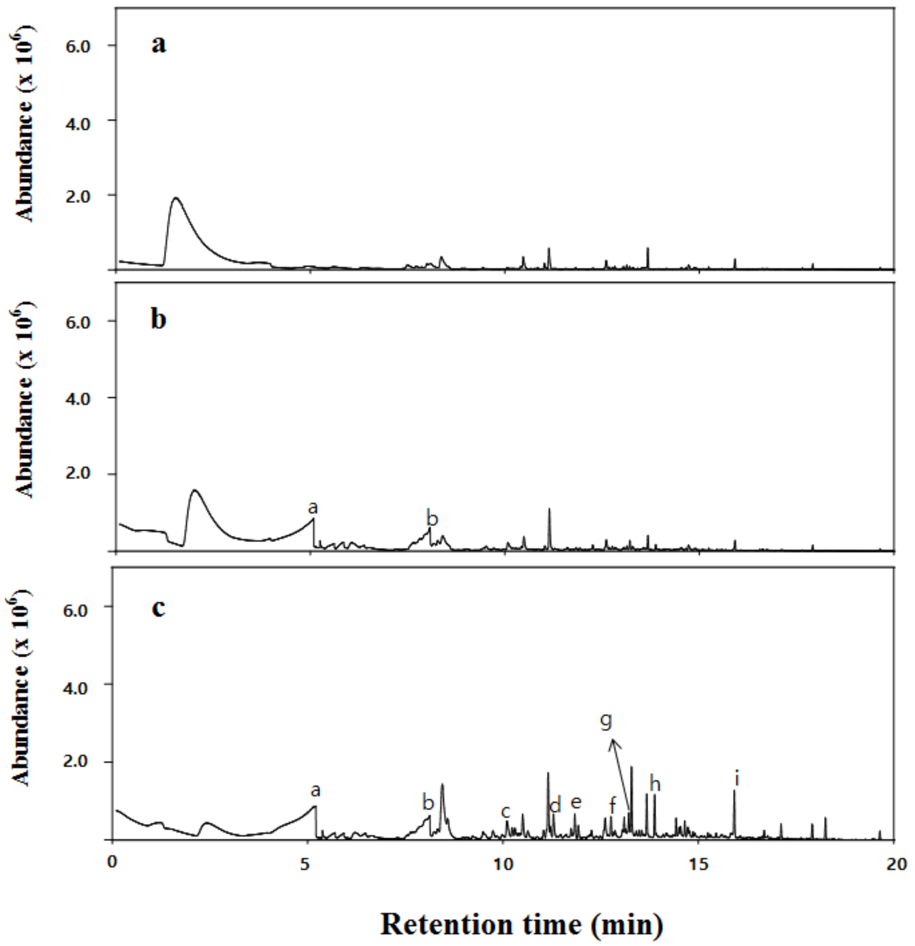

Figure S3. Detection and identification of gaseous markers indicating deterioration of oyster freshness using SPME-GC-MS. Organic compound peaks of gas samples prepared using SPME were detected from the headspace of oyster at 0 day (a), 1 day (b) and 5 days (c) after storage at 37°C, respectively.

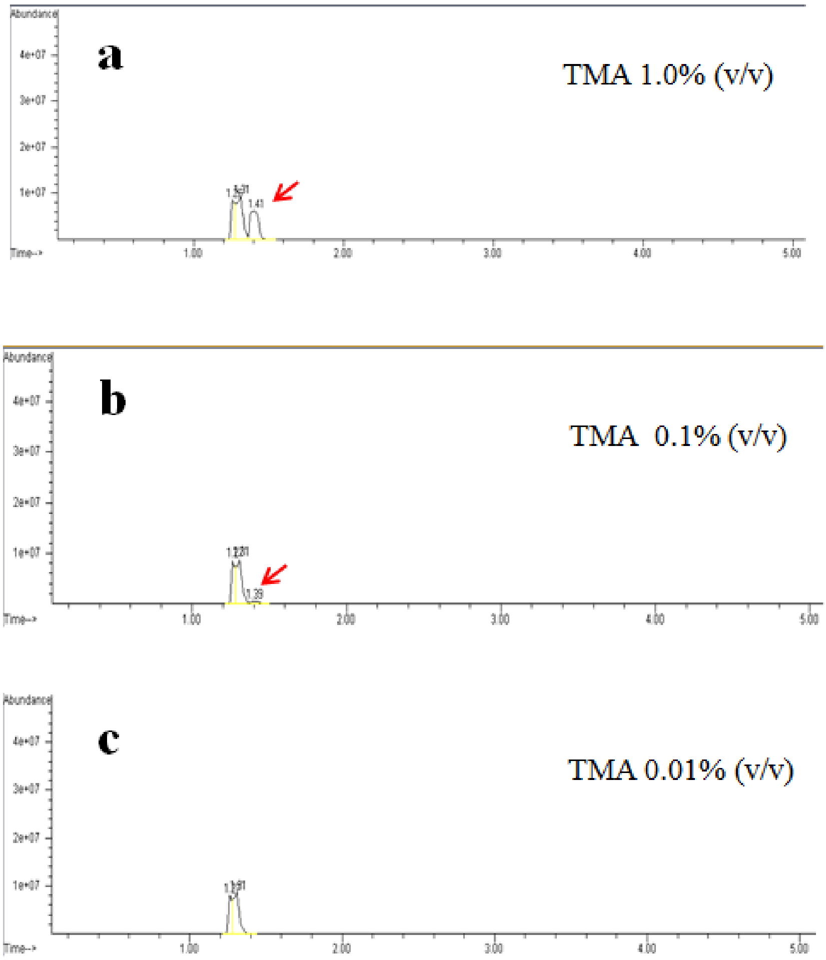

Figure S4. Detection of TMA using GC. The minimal concentration of TMA that can be detected using GC is 0.1% (b) (v/v, 1000 ppm). (c) The 0.01% (v/v, 100 ppm) of TMA was not detectable by GC.

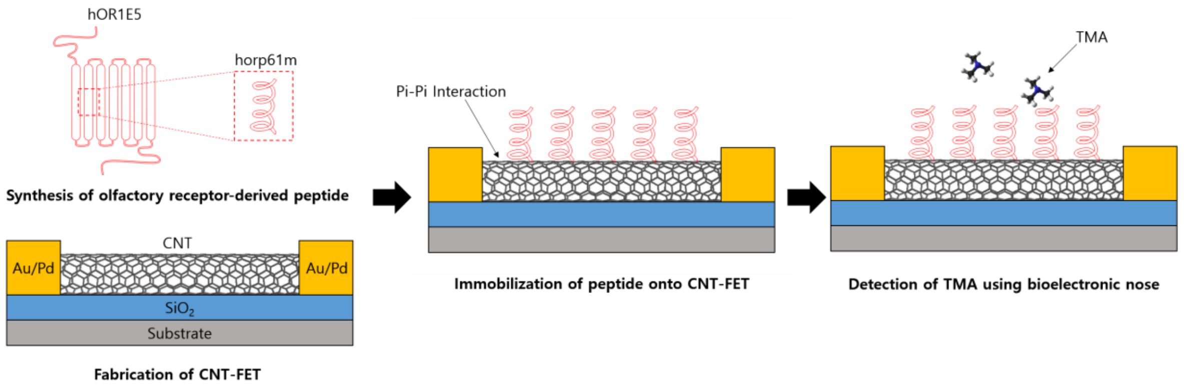

Figure S5. Fabrication and electrical measurement of bioelectronic nose.
